# Draft genome sequence of *Bacillus safensis* strain WOIS2 KX774194, a nitrile-metabolizing bacterium isolated from solid waste leachates at Olusosun dumpsite, Ojota, Lagos State, Nigeria

**DOI:** 10.1128/mra.00119-24

**Published:** 2024-06-20

**Authors:** Adewale K. Ogunyemi, Olanike M. Buraimoh, Wadzani P. Dauda, Olufunmilayo O. Akapo, Bukola C. Ogunyemi, Titilola A. Samuel, Matthew O. Ilori, Olukayode O. Amund

**Affiliations:** 1 Department of Microbiology, University of Lagos, Akoka, Lagos State, Nigeria; 2 Department of Biological Sciences (Microbiology Unit), Lagos State University of Science & Technology, Ikorodu, Lagos State, Nigeria; 3 TETFund Centre of Excellence on Biodiversity Conservation and Ecosystem Management (TCEBCEM), University of Lagos, Akoka, Lagos State, Nigeria; 4 Department of Agronomy (Crop Science Unit), Federal University, Gasuha, Yobe, Nigeria; 5 Department of Biochemistry and Microbiology, University of Zululand, KwaDlangezwa Main Campus, KwaDlangezwa, South Africa; 6 Department of Biochemistry, University of Lagos, Idi-Araba, Lagos State, Nigeria; Indiana University, Bloomington, Bloomington, Indiana, USA

**Keywords:** whole genome sequences, nitrile-metabolizing bacterium, *Bacillus safensis*, solid waste leachates, enrichment culture

## Abstract

*Bacillus safensis* strain WOIS2, a nitrile-metabolizing bacterium, was isolated from solid waste leachates at the Olusosun dumpsite, Ojota, Lagos State, Nigeria. Here, we present the draft genome sequence of strain WOIS2. These data provide valuable information on the bioprospecting of *B. safensis* nitrilase and other intriguing genes of interest.

## ANNOUNCEMENT

Nitriles are highly versatile organic compounds that can be synthesized chemically or found naturally (1). They find widespread use across multiple industries, including insecticides, plastics, solvents, synthetic rubber, and pharmaceuticals (2). Microbes producing nitrilase have attracted substantial attention from academics and entrepreneurs (3).

A bacterium that can use nitriles as its only source of carbon and nitrogen has been successfully isolated from solid waste leachates (SWL) obtained from the Olusosun dumpsite in Lagos State, Nigeria (N 6°29′21.8″; E 003°23′29.3″) (4, 5). The strain was isolated by selective enrichment culture technique. About 1.0 g of each SWL was suspended in 50 mL of MSM supplemented with nitrile (glutaronitrile, 0.2% vol/vol) in a 250 mL Erlenmeyer flask. The flasks were incubated in an orbital shaker (180 rpm) at 37°C for 7 days and further enriched. After multiple subcultures, cultures were streaked on nitrile-MS agar plates to obtain pure cultures. Strain WOIS2 was identified as *B. safensis* KX774194 (4), using the universal primers 27F and 1492R by 16S rRNA gene sequencing (6). The strain was revived on NA medium, inoculated into 5 mL of nutrient broth, and incubated for 24 hours at 37°C to obtain cell biomass. After centrifuging 2 mL of the culture at 16,000 *g* for 4 minutes, genomic DNA was extracted from the pellet using the Zymo Research Fungal/Bacterial DNA Miniprep Kit according to the manufacturer’s instructions.

The bacterial culture pellet was suspended in PBS buffer, processed in a ZR BashingBead lysis tube, and centrifuged. The supernatant was transferred to a Zymo-Spin filter, diluted with genomic lysis buffer, and then centrifuged again. DNA pre-wash buffer was added and the Zymo-Spin II CR was washed with g-DNA and wash buffer. The Zymo-Spin clean 1.5 mL was then transferred and mixed with DNA elution buffer. After centrifugation, the ultra-pure DNA was fragmented with enzymes, and size selection was conducted.

Then, xGen DNA Library Preparation Kit was used for 2 × 150 bp paired-end sequencing with the Illumina HiSequencing platform (HiSeq4000X). The obtained raw reads from each run were quality checked using FastQC (v.1.0.0, BaseSpace Illumina) and subsequently trimmed using FastXToolkit (v.2.2.5, BaseSpace Illumina) (7). Genome *de novo* assembly and assembly quality were performed using SPAdes (v.3.9.0, BaseSpace Illumina) (8, 9). The draft genome sequences were annotated using the NCBI Prokaryotic Genome Annotation Pipeline v.6.6, with default parameters (10). Also, the genome annotation and metabolic pathway identification were carried out by the RAST version 2.0 server (11). Default parameters were used except where otherwise noted.

**Fig 1:**
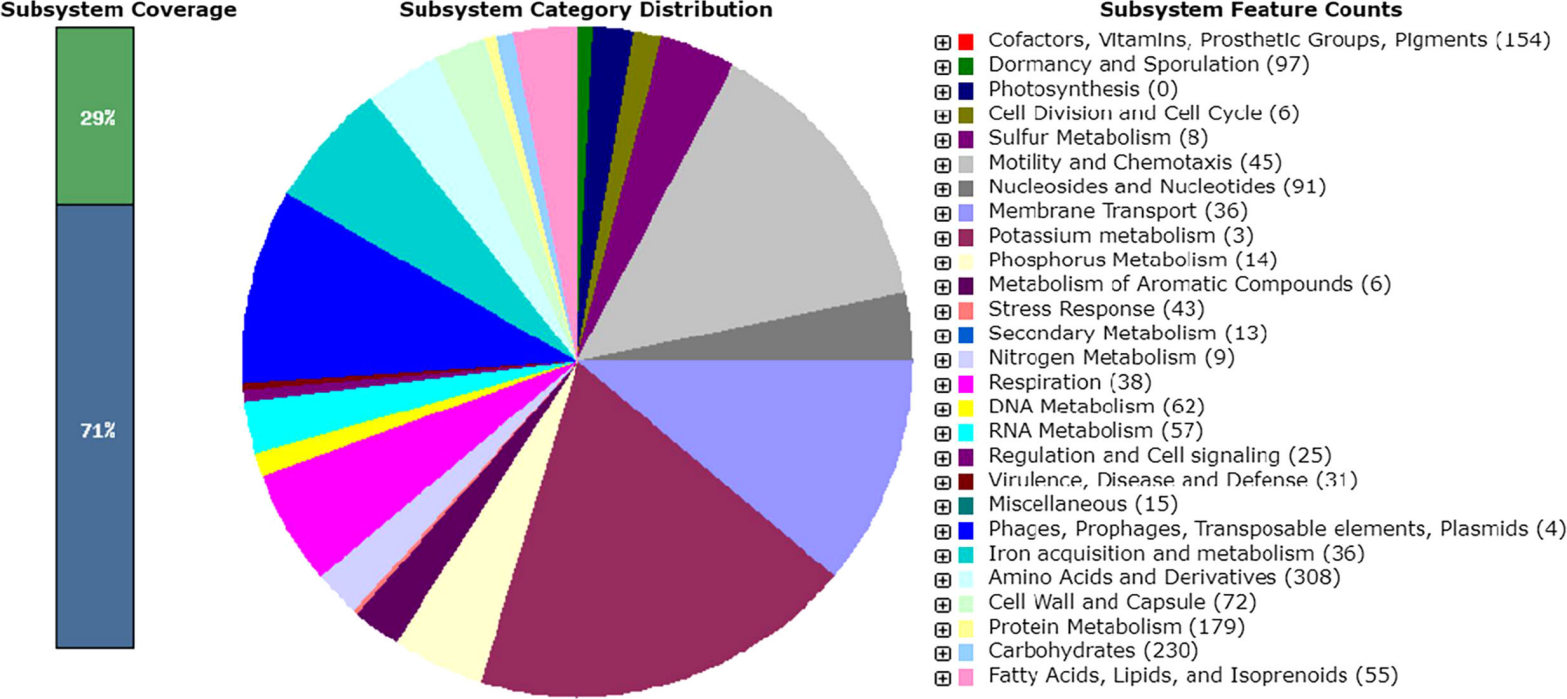
The RAST analysis-based subsystem distribution of whole genome sequence of *Bacillus safensis* strain WOIS2. Each color in the pi graph represents a particular.group of genes mentioned in the right site of the graph.

The genome features of *Bacillus safensis* strain WOIS2 are presented in Table 1. The genome of strain WOIS2 consists of 16,584,468 reads and 50 contigs, with a GC content of 41.5%. A total of 3,706,030 bp (3.7 Mb) genomic size was generated, corresponding to 429× genome coverage. Figure 1 shows the RAST analysis-based subsystem distribution of the whole genome sequence of strain WOIS2. This particular strain is undoubtedly valuable due to the presence of several intriguing genes, notably nitrilase (blr3397), which makes it highly promising for the bioremediation of nitrile-polluted environments.

**TABLE 1:** Genome features of *Bacillus safensis* strain WOIS2^*a*^

Attribute	Value
Total reads	16,584,468
Genome coverage (×)	429
Genome size (bp)	3,706,030 (3.7 Mb)
CDSs (total)	3,788
CDSs (with protein)	3,753
CDSs (without protein)	35
Genes (total)	3,875
Number of contigs	50
GC content (%)	41.5
*N*_50_	159,061 (159 kb)
*L*_50_	8
tRNA	69
rRNA	3, 9, 1
Gene (RNA)	87
ncRNA	5
Accession number	JAZAPO000000000

^*a*^ CDS, coding sequence; GC, guanine-cytosine content; tRNA, transfer RNAs; rRNA, ribosomal RNA; and ncRNA, non-coding RNAs.

## Data Availability

A whole-genome sequence of *Bacillus safensis* strain WOIS2 has been deposited in GenBank under accession number JAZAPO000000000, BioProject number PRJNA1057587, BioSample number SAMN38321375, and Sequence Read Archive (SRA) accession number SRX24013071.

## AFTER EPUB

*[This article was published on 25 June 2024 with errors in the affiliation for author Olufunmilayo O. Akapo. The affiliation was corrected in the current version, posted on 2 July 2024.]*
