# Erratum for Ogunyemi et al., “Draft genome sequence of *Bacillus safensis* strain WOIS2 KX774194, a nitrile-metabolizing bacterium isolated from solid waste leachates at Olusosun dumpsite, Ojota, Lagos State, Nigeria”

**DOI:** 10.1128/mra.00870-24

**Published:** 2024-10-10

**Authors:** Adewale K. Ogunyemi, Olanike M. Buraimoh, Wadzani P. Dauda, Olufunmilayo O. Akapo, Bukola C. Ogunyemi, Titilola A. Samuel, Matthew O. Ilori, Olukayode O. Amund

## ERRATUM

Volume 13, no. 7, e00119-24, 2024, https://journals.asm.org/doi/10.1128/mra.00119-24. Page 3, Author Affiliations: Wadzani P. Dauda’s affiliation should read “Department of Agronomy (Crop Science Unit), Federal University Gashua, Gashua, Yobe State, Nigeria.”

